# Ferrous Iron Up-regulation in Fibroblasts of Patients with Beta Propeller Protein-Associated Neurodegeneration (BPAN)

**DOI:** 10.3389/fgene.2017.00018

**Published:** 2017-02-17

**Authors:** Rosaria Ingrassia, Maurizio Memo, Barbara Garavaglia

**Affiliations:** ^1^Section of Pharmacology, Department of Molecular and Translational Medicine, University of BresciaBrescia, Italy; ^2^Molecular Neurogenetics Unit, Foundation IRCCS Neurological Institute Carlo BestaMilan, Italy

**Keywords:** Divalent metal transporter 1 (DMT1), iron, neurodegeneration, NBIA, *WDR45*, beta-propeller associated neurodegeneration (BPAN)

## Abstract

Mutations in *WDR45* gene, coding for a beta-propeller protein, have been found in patients affected by Neurodegeneration with Brain Iron Accumulation, NBIA5 (also known as BPAN). BPAN is a movement disorder with Non Transferrin Bound Iron (NTBI) accumulation in the basal ganglia as common hallmark between NBIA classes (Hayflick et al., 2013). WDR45 has been predicted to have a role in autophagy, while the impairment of iron metabolism in the different NBIA subclasses has not currently been clarified. We found the up-regulation of the ferrous iron transporter (-)IRE/Divalent Metal Transporter1 and down-regulation of Transferrin receptor in the fibroblasts of two BPAN affected patients with splicing mutations 235+1G>A (BPAN1) and 517_519ΔVal 173 (BPAN2). The BPAN patients showed a concomitant increase of intracellular ferrous iron after starvation. An altered pattern of iron transporters with iron overload is highlighted in BPAN human fibroblasts, supporting for a role of DMT1 in NBIA. We here present a novel element, about iron accumulation, to the existing knowledge in field of NBIA. Attention is focused to a starvation-dependent iron overload, possibly accounting for iron accumulation in the basal ganglia. Further investigation could clarify iron regulation in BPAN.

## Introduction

*De novo* mutations in WDR45 gene on chromosome Xp11 have been found in patients with BPAN (Hayflick et al., 2013), a movement disorder with iron accumulation in the basal ganglia characterized by early childhood psychomotor retardation remaining static until the third decade of life, after which time affected individuals develop progressive dystonia-Parkinsonism and dementia (Haack et al., 2012, 2013; Lunt et al., 2013; Saitsu et al., 2013; Schneider et al., 2013). BPAN is always sporadic, with a female preponderance indicative of X-linked dominant inheritance with lethality in males. The identified allelic mutations of WDR45 produce loss of function and impairment of autophagy as principal knowledge of BPAN neurodegeneration, because the role of iron metabolism and cerebral iron deposition in the disease is currently not clarified.

WDR45 is a member of the WD40 repeat protein family. WD40 domains are units of conserved 40 aminoacids with a consensus repeat of tryptophan-aspartic acid (WD) residues for interaction with phospholipids. WD40 proteins have a highly symmetrical, seven-bladed, beta-propeller platform structure, coordinating protein–protein interactions. In particular, WDR45 protein that is regulated by the induction of autophagy, has been proposed as a biomarker of autophagosome formation (Tsuyuki et al., 2014). Autophagy is a cellular degradation system for long-lived proteins and organelles, activated during nutrient starvation with the contribution of the ATG genes, yeast autophagy-related genes. Furthermore, the WIPI4/WDR45 gene of the WIPI (WD repeat protein interacting with phosphoinositides) family, is a mammalian ortholog of the yeast autophagy gene ATG18, particularly induced during autophagy. Although the mechanistic relationship between WDR45 deficiency and the causes of BPAN neurodegeneration are unknown, a clear pattern of clinical imaging and natural history data leads to the identification of the specific phenotype of patients. This NBIA disorder was indeed called ‘beta-propeller protein-associated neurodegeneration’ (BPAN) (Haack et al., 2012).

Recently, we found iron and DMT1 accumulation in the substantia nigra (SN) of a mice model of neurodegeneration with Parkinsonism, the NF-kB/c-rel knockout mice (Baiguera et al., 2012), according to previous findings in Parkinson’s patients (Salazar et al., 2008), and during the early phase of brain ischemia (Ingrassia et al., 2012). Therefore, we hypothesized a relationship between *de novo* mutations in WDR45 gene and the isoform without Iron Response Element (IRE) of ferrous iron transporter DMT1, (-)IRE/DMT1. We based this work on the well-acknowledged evidence that human primary fibroblasts efficiently reflect molecular and functional changes associated to neurodegenerative pathologies (Campanella et al., 2012; Zanellati et al., 2015). To this purpose, we studied the pattern of iron transporters and ferrous iron in primary fibroblasts of two BPAN patients to assess whether the impairment of iron transport could account for its accumulation. DMT1 function is associated to a complex structure and its regulation is finely tuned by the expression of four different isoforms, generated by two alternative splicings (Hubert and Hentze, 2002; Garrick et al., 2006; Mackenzie et al., 2007). The first splicing produces two different promoter regions, 1A and 1B. The 1A splicing is responsive to hypoxia in rat PC12 cells (Lis et al., 2005) and HIF-2 alpha in Caco-2/TC7 cells (Mastrogiannaki et al., 2009), while the 1B isoform is responsive to NF-kB in P19 mouse embryonic carcinoma cells and mouse primary cortical neurons (Paradkar and Roth, 2006a,b; Ingrassia et al., 2012). 1B isoform is also responsive to HIF-1 alpha in HepG2 cells (Wang et al., 2010; Qian et al., 2011). The second splicing implies that both 1A and 1B isoforms may have or not an IRE at the opposite 3’ untranslated region. This mechanism is sensitive to feedback regulation by intracellular iron levels (Hentze and Kühn, 1996; Pantopoulos, 2004; Recalcati et al., 2010; Sanchez et al., 2011; Wilkinson and Pantopoulos, 2014). In particular, the mRNA analysis of 1B/(+)IRE isoform shows the predicted down-regulation in conditions of intracellular iron overload, as well as TfR, while the 1B/(-)IRE isoform can be regulated by iron-independent mechanism (Hubert and Hentze, 2002). While the specific expression of 1B/(+)IRE isoform and 1A/(+)IRE is also shown in primary rat hippocampal neurons and astrocytes, respectively (Pelizzoni et al., 2012, 2013), only 1A/(+)IRE over-expression showed competence for ferrous iron uptake. Indeed, intracellular iron overload leads to the canonical IRE/IRP post-transcriptional control with down-regulation of both TfR and (+)IRE/DMT1 isoform, like several mRNA encoding proteins of iron, oxygen and energy metabolism (Hubert and Hentze, 2002; Pantopoulos, 2004; Wilkinson and Pantopoulos, 2014). In this respect, while (+)IRE/DMT1 isoform shouldn’t contribute to the increased uptake of ferrous iron during iron overload, (-)IRE/DMT1 isoform is not influenced by intracellular iron perturbation. Conversely, (-)IRE/DMT1 could be up-regulated at transcriptional (Paradkar and Roth, 2006a,b; Ingrassia et al., 2012) or post-translational level, via proteasome impairment (Garrick et al., 2012). Importantly, not only (-)IRE/DMT1 is independent from post-transcriptional iron regulation with the role in Non Transferrin Bound Iron (NTBI) internalization, but it is also involved in the life-sustaining TfR cycle (Tabuchi et al., 2010). In fact, it is localized on early endosomes, with TfR co-localization during recycling. Moreover, a different subcellular distribution of DMT1 is reported with cell membrane, cytoplasmic and nuclear localization (Roth et al., 2000; Lis et al., 2004). Since iron is essential for cellular homeostasis, its intracellular level and transport has to be tightly controlled due to the damaging role in the Haber–Weiss/Fenton autocatalytic reactions, thus supporting for a role of (-)IRE/DMT1 in NBIA. Indeed, (-)IRE/DMT1 could play a peculiar role in the increase of the ferrous iron in BPAN and, more generally, in the other forms NBIA. Iron accumulation in the basal ganglia represents a common hallmark between the different classes of NBIA (Gregory and Hayflick, 2013; Rouault, 2013; Levi and Finazzi, 2014; Hogarth, 2015; Nishioka et al., 2015; Arber et al., 2016), and is a central element for a more innovative therapy (Zorzi and Nardocci, 2013). In BPAN patients, where the impairment of autophagy is the principal peculiarity, due to mutations of WDR45 gene, involved in autophagosome maturation (Arber et al., 2016 and references therein), besides iron accumulation, we highlight a possible relationship between the impairment of iron homeostasis and the altered pattern of DMT1 and TfR with a consequent ferrous iron overload. Since no evidence up to now is present about a derangement of iron metabolism in BPAN patients, we analyzed primary fibroblasts of BPAN patients to assess whether they could show a parallel pattern to cerebral iron overload. We thus hypothesized that, in BPAN patients, (-)IRE/DMT1, the isoform unresponsive to IRE/IRP post-transcriptional regulation, could be up-regulated, as a possible mediator of NTBI, at least in proliferating cells like fibroblasts.

## Materials and Methods

### Cell Culture and Treatment

Human primary fibroblasts from two BPAN affected patients and healthy subjects of the same age, gender and subcultures, derived from skin biopsy, were kindly provided from the “Cell line and DNA Bank of Genetic Movement Disorders and Mitochondrial Diseases” of the Telethon Network of Genetic Biobank and were cultured in DMEM supplemented with 10% FBS, L-glutamine and antibiotic solution in a 5% CO_2_ incubator. After plating of 2 × 10^4^ cells/cm^2^ in 10 cm^2^ petri dishes (Corning) and overnight growth in complete DMEM, the cells were exposed or not to starvation for 2 h, in DMEM, L-Glutamine and antibiotic solution, without FBS, in a 5% CO_2_ incubator and then collected for cellular extracts preparation.

### Immunoblotting

Total protein extracts from human fibroblasts were prepared for Immunoblot analyses. Cells were lysed with lysis buffer (50 mM Tris-HCl, pH 7.5, 150 mM NaCl, 1 mM EDTA, 1% Triton X-100, Complete EDTA-free protease inhibitor cocktail and Phospho stop reagent, Roche). Cell lysates were clarified by centrifugation at 12,000*g* for 20 min and analyzed by SDS-PAGE and immunoblotting, against the Protein Molecular Weight Marker Odyssey (LI-COR). The following antibodies were used: rabbit anti-rat DMT1(-)IRE (Alpha Diagnostic International, NRAMP23-A, affinity pure IgG), mouse anti-human TfR (Invitrogen, 13–6800), mouse anti-GAPDH (Millipore, AB2302). Signal detection and densitometric normalization of protein expression were performed on three experiments in triplicate, by LI-COR/Odyssey Infra-red analysis.

### Turnbull’s Staining

Turnbull’s staining for ferrous iron was performed according to Park et al. (2015). Briefly, cells were plated onto poly-L-lysine-coated coverslips at 2 × 10^4^ cells/cm^2^ in 2 cm^2^ multi-wells petri dishes (Corning) and overnight grown. After 2 h starvation in DMEM serum free with L-Glutamine, the fibroblasts were washed in phosphate buffer saline, fixed in 4% paraformaldehyde 30 min and washed in phosphate buffer saline. Then, incubation with 2% potassium ferricyanide in 2%HCl, 30 min was performed followed by three washes in distilled water. Iron staining was observed under Olympus inverted microscope in phase contrast using a 20X PanApoN lens. Normalization was based on cellular number. Quantification was performed by ImageJ (National Institute of Health) on three experiments in triplicate, which are normalized by size of the selected area scale setting in calibrated units of the analyzed cellular field.

### Statistical Analysis

Experimental differences between the groups were calculated as the means ± SE and subjected to the analysis of variance (ANOVA) with Bonferroni *post hoc* test, using Graph Pad Prism software (version 4.0). All differences were considered statistically significant at the *P*-value <0,05.

## Results

**FIGURE 1:**
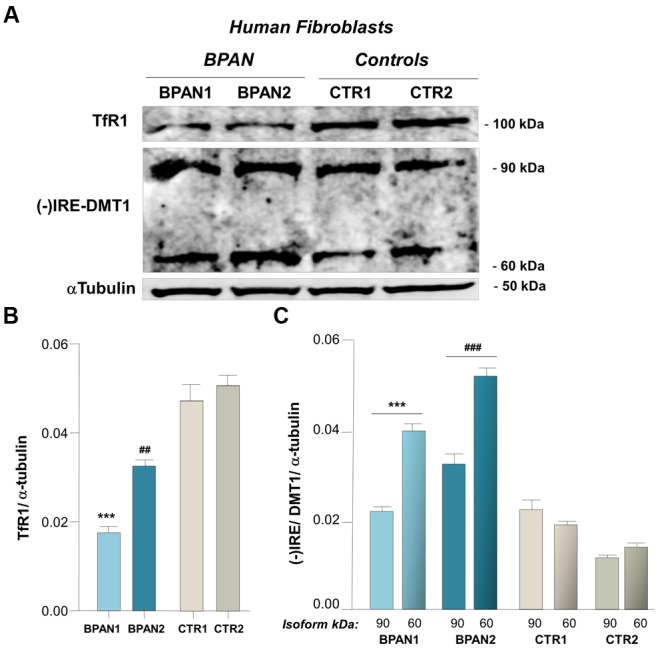
**(-)IRE/DMT1 isoform and TfR expression in human fibroblast of BPAN patients. (A)** Representative western blots of (-)IRE/DMT1 and TfR in human fibroblasts of BPAN patients respect to health controls. **(B)** Densitometric analysis shows TfR protein level respect to controls (^∗∗∗^*p* < 0,001 and ^##^*p* < 0,01, BPAN 1 and BPAN2, respectively, vs. Controls) and **(C)** (-)IRE/DMT1 protein level in BPAN1 and BPAN2 respect to control fibroblasts, CTR1 and CTR2 (^∗∗∗^*p* < 0,001 BPAN1 and ^###^*p* < 0,001 BPAN2, 60 and 90 kDa components, vs. Controls). As housekeeping internal control α-tubulin was evaluated.

**FIGURE 2:**
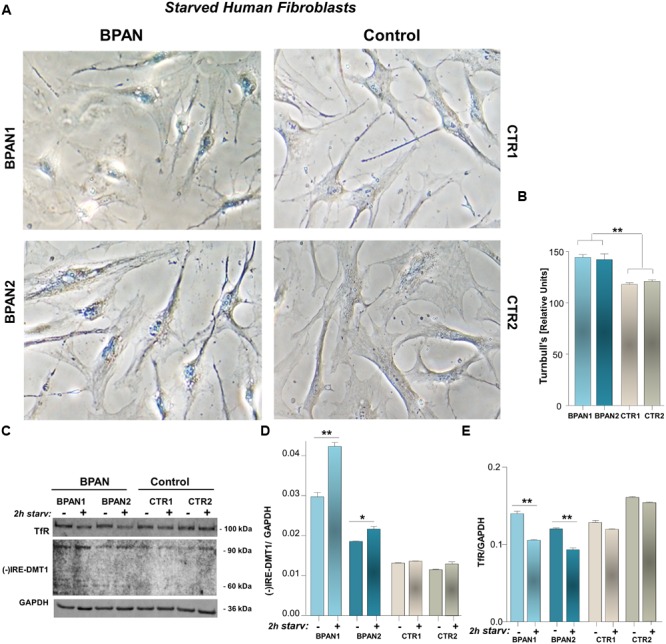
**Ferrous iron in BPAN fibroblasts under starvation, respect to (-)IRE/DMT1 and TfR. (A)** Iron enrichment was evaluated by Turnbull’s staining, after 2 h starvation of BPAN and control fibroblasts, and showed in a representative detection. **(B)** Staining intensity was determined by ImageJ as saturation signal of the whole cellular field normalized for cellular density. Turnbull’s staining is showed as the percentage of the Relative Units signal of BPAN vs. Controls, normalized by size of the selected area scale setting in calibrated units of the analyzed cellular field (^∗∗^*p* < 0,01 BPAN fibroblasts respect to Controls). **(C)** Representative western blot of (-)IRE/DMT1 and TfR in human fibroblast of BPAN patients under starvation, respect to starved controls. **(D,E)** Densitometric analysis after starvation shows the 90 kDa isoform of (-)IRE/DMT1 and TfR protein level in BPAN1 and BPAN2, respect to control fibroblasts (^∗∗^*p* < 0,001, ^∗^*p* < 0,01 BPAN 1 and BPAN2 under starvation vs. relative Controls). As housekeeping internal control GAPDH was evaluated.

We investigated (-)IRE/DMT1 and TfR expression in human fibroblasts of two BPAN affected patients, BPAN1 and BPAN2, respect to fibroblasts of control healthy subjects, CTR1 and CTR2. (-)IRE/DMT1, the isoform unresponsive to IRE/IRP-dependent post-transcriptional regulation, and TfR showed a significantly altered pattern of expression. We found significant up-regulation, between two and fourfold, of (-)IRE/DMT1 in fibroblasts of BPAN1 and BPAN2 patients, compared to CTR1 and CTR2 (^∗∗∗^*p* < 0,001 BPAN1 and ^###^*p* < 0,001 BPAN2 vs. CTR1 and CTR1) as showed in a representative immunoreactivity experiment (**Figures 1A,C**). Interestingly, both (-)IRE/DMT1 glycosylated components were up-regulated: the 60 kDa component, the one early glycosylated, was more significantly up-regulated than the 90 kDa fully glycosylated one. Although we performed immunoreactivity experiments with an isoform-specific antibody against (-)IRE/DMT1, we then verified it subjecting BPAN and Control fibroblasts to the same analysis at different passages of growth, as shown in Supplemental Figures 1A,B. We found reproducible levels of detection, according to the recognized variability of immunoreactivity against the 60 and 90 kDa differentially glycosylated components. We conversely found the concomitant down-regulation of TfR in the fibroblasts of BPAN patients (^∗∗∗^*p* < 0,001 and ^##^*p* < 0,01 respect to Controls; **Figures 1A,B**), in accordance with the canonical IRE/IRP post-transcriptional regulation during intracellular iron overload. Ferrous iron was significantly increased, after 2 h of starvation, as shown by Turnbull’s staining, in human fibroblasts from BPAN1 and BPAN2 affected patients, with respect to control subjects CTR1 and CTR2, (^∗∗^*p* < 0,01 vs. Controls; **Figures 2A,B**). No significant differences were present in untreated fibroblasts (data not shown). Concomitant to the increase of ferrous iron in BPAN fibroblasts, after starvation we found the up-regulation of (-)IRE/DMT1 protein compared to relative basal levels in BPAN patients (^∗∗^*p* < 0,001 and ^∗^*p* < 0,01: BPAN1 and BPAN2, respectively, vs. relative Controls; **Figures 2C,D**). On the other hand, TfR appears down-regulated under starvation in BPAN fibroblasts extracts in respect to the relative basal levels (^∗∗^*p* < 0,001: BPAN1 and BPAN2 vs. relative Controls; **Figures 2C,E**).

## Discussion

As expected, we found the up-regulation of (-)IRE/DMT1 with down-regulation of TfR in the fibroblasts of BPAN patients respect to health controls (**Figures 1A–C**) in accordance with an intracellular milieu of iron overload. In particular, the early glycosylated 60 kDa component of DMT1 (Tabuchi et al., 2002) was more significantly up-regulated in these patients, supporting for a rapid positive feedback of the early glycosylated product, possibly as a downstream effect of the known response of endoplasmic reticulum to the impairment of autophagy (Khaminets et al., 2015). Moreover, a significant increase of Turnbull’s staining for intracellular ferrous iron was present in BPAN fibroblasts under starvation (**Figures 2A,B**). As nutrient deprivation physiologically induces autophagy, we could infer that this treatment in BPAN fibroblasts, where autophagy is impaired, could lead to reduced lysosomal degradation with accumulation of (-)IRE/DMT1 and the consequent increase of ferrous iron. It is in fact well acknowledged that, under starvation, autophagy determines degradation of unneeded proteins in order to activate anabolic homeostasis, as a consequence of the increased intracellular pool of amino acids (Mizushima et al., 2004; Chen et al., 2014; Kaur and Debnath, 2015). In this respect, autophagy not only sustains the turnover of cellular components, but also the regulation of metabolism, membrane transport and host defenses (Boya et al., 2013). Indeed, compromised autophagy in BPAN cells could affect the removal of toxic proteins, like the ferrous iron transporter, thus highlighting a pharmacological potential target (Rubinsztein et al., 2015). In fact, during starvation, when autophagy has an essential role in the balance of anabolic/catabolic homeostasis and its impairment could be detrimental, we observed the significant increase of Turnbull’s staining for ferrous iron in BPAN fibroblasts respect to controls (**Figures 2A,B**). Concomitantly, we found the up-regulation of (-)IRE/DMT1 in respect to relative basal level (**Figures 2C,D**), in the presence of a significant down-regulation of TfR, during starvation (**Figures 2C,E**). These evidences support a significant increase in the transport and recycling of ferrous iron, compared to the ferric component, with possible impairment of the life sustaining TfR cycle and the intracellular redox potential in relationship with the Haber–Weiss and Fenton reaction. Moreover, the starvation-dependent TfR down-regulation reflects the IRE-IRP post-translational regulation, due to concomitant ferrous iron up-regulation, as shown by Turnbull’s staining. While this work was in progress, DMT1 up-regulation was described in the brain of PLAG26 knockout mouse model (Beck et al., 2015), a different NBIA subclass, as a first evidence focusing the attention to the ferrous iron transporter. Interestingly, Beck et al. (2015) show the up-regulation of (+)IRE/DMT1 in PLAG26 knockout mice model, in the central nervous system, as well as the concomitant TfR up-regulation with cerebral iron overload. This is in line with findings on a different form of neurodegeneration with iron overload as in Parkinson disease (Salazar et al., 2008), where, in the SN of post-mortem patients, iron levels were increased with (+)IRE/DMT1 up-regulation. Conversely, the different pattern of expression we showed in BPAN fibroblasts, with down-regulation of TfR, represents a condition of iron overload, according to IRE-IRP regulation, confirmed by ferrous iron staining. In this cellular contest, concomitant down-regulation of (+)IRE/DMT1 is also expected. Although in apparent opposition with respect to the evidences in PLAG26 knockout mice and Parkinson’s patients, the results we reported in primary fibroblasts could account for the evaluation of proliferating vs. post-mitotic cell types. The significant findings we obtained in BPAN fibroblasts highlight the status of iron transporters, with up-regulation of (-)IRE/DMT1 associated to increased uptake of ferrous iron. Furthermore, the up-regulation of (-)IRE/DMT1 in BPAN fibroblasts could account for a downstream impairment of proteasome degradation, possibly due to impairment of autophagy, consequent to WDR45 *de novo* mutations. We here highlight the underpinning relationship between altered control of iron homeostasis and impaired autophagy in BPAN. In fact, we found an altered pattern of both (-)IRE/DMT1 and TfR transporters in fibroblasts of BPAN patients, both at basal level and under starvation, autophagy-dependent, that lead us to hypothesize an imbalance in the uptake of redox iron species. This finding was further supported by the increased ferrous iron in BPAN fibroblasts under starvation, as found in Turnbull’s staining. These evidences shed light on the role of iron metabolism as an important player downstream to autophagy impairment, under failure of specific metabolic responses to nutrient deprivation, due to WDR45 mutations. In this respect, we need to consider that the control of iron homeostasis is essential for healthy cellular life and that the intracellular delivery of iron has to be tightly regulated because of its possible damaging role in the Haber–Weiss/Fenton autocatalytic reactions. Importantly, the altered pattern of iron transporters with iron overload we showed in BPAN human fibroblasts lead us to emphasize a relationship with the general hallmark of NTBI in NBIA (Hayflick et al., 2013).

## Ethics Statement

Cell line and DNA Bank of Genetic Movement Disorders and Mitochondrial Diseases” of the Telethon Network of Genetic Biobank Human primary fibroblasts from two BPAN affected patients and healthy subjects of the same age, gender and subcultures, derived from skin biopsy (http://biobanknetwork.telethon.it/pages/view/thecharter#ethical_guidelines_of_tngb). The subjects of this study where two female patients affected by Beta-propeller protein associated Neurodegeneration (BPAN).

## Author Contributions

Research project conception: RI. Research project organization: RI, BG, and MM. Research project execution: RI. Manuscript writing of the draft: RI. Manuscript review and critique: BG, RI, and MM.

## Conflict of Interest Statement

The authors declare that the research was conducted in the absence of any commercial or financial relationships that could be construed as a potential conflict of interest.

## References

[B1] Arber C. E. Li A. Houlden H. Wray S. (2016). Insights into molecular mechanisms of disease in neurodegeneration with brain iron accumulation: unifying theories. *Neuropathol. Appl. Neurobiol.* 42 220–241. 10.1111/nan.12242 25870938 PMC4832581

[B2] Baiguera C. Alghisi M. Pinna A. Bellucci A. De Luca M. A. Frau L. (2012). Late-onset Parkinsonism in NFκB/c-Rel-deficient mice. *Brain* 135(Pt 9) 2750–2765. 10.1093/brain/aws193 22915735 PMC3437025

[B3] Beck G. Shinzawa K. Hayakawa H. Baba K. Yasuda T. Sumi-Akamaru H. (2015). Deficiency of calcium-independent phospholipase A2 Beta induces brain iron accumulation through upregulation of divalent metal transporter 1. *PLoS ONE* 10:e0141629 10.1371/journal.pone.0141629 PMC4624760 26506412

[B4] Boya P. Reggiori F. Codogno P. (2013). Emerging regulation and functions of autophagy. *Nat. Cell Biol.* 15 713–720. 10.1038/ncb2788 23817233 PMC7097732

[B5] Campanella D. Privitera M. Guaraldo E. Rovelli C. Barzaghi B. Garavaglia P. (2012). Skin fibroblasts from pantothenate kinase-associated neurodegeneration patients show altered cellular oxidative status and have defective iron-handling properties. *Hum. Mol. Genet.* 21 4049–4059. 10.1093/hmg/dds229 22692681

[B6] Chen R. Zou Y. Mao D. Sun D. Gao G. Shi J. (2014). The general amino acid control pathway regulates mTOR and autophagy during serum/glutamine starvation. *J. Cell Biol.* 206 173–182. 10.1083/jcb.201403009 25049270 PMC4107793

[B7] Garrick M. D. Kuo H. C. Vargas F. Singleton S. Zhao L. Smith J. J. (2006). Comparison of mammalian cell lines expressing distinct isoforms of divalent metal transporter 1 in a tetracycline-regulated fashion. *Biochem. J.* 398 539–546. 10.1042/BJ20051987 16737442 PMC1559468

[B8] Garrick M. D. Zhao L. Roth J. A. Jiang H. Feng J. Foot N. J. (2012). Isoform specific regulation of divalent metal transporter (DMT1) by proteasomal degradation. *Biometals* 25 787–793. 10.1007/s10534-012-9522-1 22310887 PMC3493171

[B9] Gregory A. Hayflick S. (2013). “Neurodegeneration with brain iron accumulation disorders overview,” in *GeneReviews* eds Pagon R. A. Adam M. P. Ardinger H. H. Wallace S. E. Amemiya A. Bean L. J. H. (Seattle, WA: University of Washington, Seattle).

[B10] Haack T. B. Hogarth P. Gregory A. Prokisch H. Hayflick S. J. (2013). BPAN: the only X-linked dominant NBIA disorder. *Int. Rev. Neurobiol.* 110 85–90. 10.1016/B978-0-12-410502-7.00005-3 24209435

[B11] Haack T. B. Hogarth P. Kruer M. C. Gregory A. Wieland T. Schwarzmayr T. (2012). Exome sequencing reveals de novo WDR45 mutations causing a phenotypically distinct. X-linked dominant form of NBIA. *Am. J. Hum. Genet.* 7 1144–1149. 10.1016/j.ajhg.2012.10.019 PMC3516593 23176820

[B12] Hayflick S. J. Kruer M. C. Gregory A. Haack T. B. Kurian M. A. Houlden H. H. (2013). β-Propeller protein-associated neurodegeneration: a new X-linked dominant disorder with brain iron accumulation. *Brain* 136(Pt 6) 1708–1717. 10.1093/brain/awt095 23687123 PMC3673459

[B13] Hentze M. W. Kühn L. C. (1996). Molecular control of vertebrate iron metabolism: mRNA-based regulatory circuits operated by iron, nitric oxide, and oxidative stress. *Proc. Natl. Acad. Sci. U.S.A*. 6 8175–8182. 10.1073/pnas.93.16.8175 PMC38642 8710843

[B14] Hogarth P. (2015). Neurodegeneration with brain iron accumulation: diagnosis and management. *J. Mov. Disord.* 8 1–13. 10.14802/jmd.14034 25614780 PMC4298713

[B15] Hubert N. Hentze M. W. (2002). Previously uncharacterized isoforms of divalent metal transporter (DMT)-1: implications for regulation and cellular function. *Proc. Natl. Acad. Sci. U.S.A.* 99 12345–12350. 10.1073/pnas.192423399 12209011 PMC129447

[B16] Ingrassia R. Lanzillotta A. Sarnico I. Benarese M. Blasi F. Borgese L. (2012). 1B/(-)IRE DMT1 expression during brain ischemia contributes to cell death mediated by NF-κB/RelA acetylation at Lys310. *PLoS ONE* 7:e38019 10.1371/journal.pone.0038019 PMC3362534 22666436

[B17] Kaur J. Debnath J. (2015). Autophagy at the crossroads of catabolism and anabolism. *Nat. Rev. Mol. Cell Biol.* 16 461–472. 10.1038/nrm4024 26177004

[B18] Khaminets A. Heinrich T. Mari M. Grumati P. Huebner A. K. Akutsu M. (2015). Regulation of endoplasmic reticulum turnover by selective autophagy. *Nature* 522 354–358. 10.1038/nature14498 26040720

[B19] Levi S. Finazzi D. (2014). Neurodegeneration with brain iron accumulation: update on pathogenic mechanisms. *Front. Pharmacol.* 5:99 10.3389/fphar.2014.00099 PMC4019866 24847269

[B20] Lis A. Barone T. A. Paradkar P. N. Plunkett R. J. Roth J. A. (2004). Expression and localization of different forms of DMT1 in normal and tumor astroglial cells. *Brain Res. Mol. Brain Res*. 17 62–70. 10.1016/j.molbrainres.2003.11.023 14992816

[B21] Lis A. Paradkar P. N. Singleton S. Kuo H. C. Garrick M. D. Roth J. A. (2005). Hypoxia induces changes in expression of isoforms of the divalent metal transporter (DMT1) in rat pheochromocytoma (PC12) cells. *Biochem. Pharmacol.* 69 1647–1655. 10.1016/j.bcp.2005.03.023 15896344

[B22] Lunt P. Carey M. Hardy J. Meitinger T. Prokisch H. Hogarth P. (2013). β-Propeller protein-associated neurodegeneration: a new X-linked dominant disorder with brain iron accumulation. *Brain* 136(Pt 6) 1708–1717. 10.1093/brain/awt095 23687123 PMC3673459

[B23] Mackenzie B. Takanaga H. Hubert N. Rolfs A. Hediger M. A. (2007). Functional properties of multiple isoforms of human divalent metal-ion transporter 1 (DMT1). *Biochem. J.* 403 59–69. 10.1042/BJ20061290 17109629 PMC1828886

[B24] Mastrogiannaki M. Matak P. Keith B. Simon M. C. Vaulont S. Peyssonnaux C. (2009). HIF-2alpha, but not HIF-1alpha, promotes iron absorption in mice. *J. Clin. Invest.* 119 1159–1166. 10.1172/JCI38499 19352007 PMC2673882

[B25] Mizushima N. Yamamoto A. Matsui M. Yoshimori T. Ohsumi Y. (2004). In vivo analysis of autophagy in response to nutrient starvation using transgenic mice expressing a fluorescent autophagosome marker. *Mol. Biol. Cell* 15 1101–1111. 10.1091/mbc.E03-09-0704 14699058 PMC363084

[B26] Nishioka K. Oyama G. Yoshino H. Li Y. Matsushima T. Takeuchi C. (2015). High frequency of beta-propeller protein-associated neurodegeneration (BPAN) among patients with intellectual disability and young-onset parkinsonism. *Neurobiol. Aging* 36 e9–e2004. 10.1016/j.neurobiolaging.2015.01.020 25744623

[B27] Pantopoulos K. (2004). Iron metabolism and the IRE/IRP regulatory system: an update. *Ann. N. Y. Acad. Sci.* 1012 1–13. 10.1196/annals.1306.001 15105251

[B28] Paradkar P. N. Roth J. A. (2006a). Post-translational and transcriptional regulation of DMT1 during P19 embryonic carcinoma cell differentiation by retinoic acid. *Biochem. J.* 394(Pt 1) 173–183. 16232120 10.1042/BJ20051296 PMC1386015

[B29] Paradkar P. N. Roth J. A. (2006b). Nitric oxide transcriptionally down-regulates specific isoforms of divalent metal transporter (DMT1) via NF-kappaB. *J. Neurochem.* 96 1768–1777. 16539692 10.1111/j.1471-4159.2006.03702.x

[B30] Park S. Kwak B. K. Jung J. (2015). Sensitivity of susceptibility-weighted imaging in detecting superparamagnetic iron oxide-labeled mesenchymal stem cells: a comparative study. *Iran J. Radiol.* 12:e20782 10.5812/iranjradiol.20782 PMC4389178 25901258

[B31] Pelizzoni I. Zacchetti D. Campanella A. Grohovaz F. Codazzi F. (2013). Iron uptake in quiescent and inflammation-activated astrocytes: a potentially neuroprotective control of iron burden. *Biochim. Biophys. Acta* 1832 1326–1333. 10.1016/j.bbadis.2013.04.007 23583428 PMC3787737

[B32] Pelizzoni I. Zacchetti D. Smith C. P. Grohovaz F. Codazzi F. (2012). Expression of divalent metal transporter 1 in primary hippocampal neurons: reconsidering its role in non-transferrin-bound iron influx. *J. Neurochem.* 120 269–278. 10.1111/j.1471-4159.2011.07578.x 22121954

[B33] Qian Z. M. Wu X. M. Fan M. Yang L. Du F. Yung W. H. (2011). Divalent metal transporter 1 is a hypoxia-inducible gene. *J. Cell. Physiol.* 226 1596–1603. 10.1002/jcp.22485 20945371

[B34] Recalcati S. Minotti G. Cairo G. (2010). Iron regulatory proteins: from molecular mechanisms to drug development. *Antioxid. Redox. Signal.* 15 1593–1616. 10.1089/ars.2009.2983 20214491

[B35] Roth J. A. Horbinski C. Feng L. Dolan K. G. Higgins D. Garrick M. D. (2000). Differential localization of divalent metal transporter 1 with and without iron response element in rat PC12 and sympathetic neuronal cells. *J. Neurosci.* 15 7595–7601. 10.1523/JNEUROSCI.20-20-07595.2000 PMC6772877 11027219

[B36] Rouault T. A. (2013). Iron metabolism in the CNS: implications for neurodegenerative diseases. *Nat. Rev. Neurosci.* 14 551–564. 10.1038/nrn3453 23820773

[B37] Rubinsztein D. C. Bento C. F. Deretic V. (2015). Therapeutic targeting of autophagy in neurodegenerative and infectious diseases. *J. Exp. Med.* 212 979–990. 10.1084/jem.20150956 26101267 PMC4493419

[B38] Saitsu H. Nishimura T. Muramatsu K. Kodera H. Kumada S. Sugai K. (2013). De novo mutations in the autophagy gene WDR45 cause static encephalopathy of childhood with neurodegeneration in adulthood. *Nat. Genet.* 45 445–9 449e1 10.1038/ng.2562 23435086

[B39] Salazar J. Mena N. Hunot S. Prigent A. Alvarez-Fischer D. Arredondo M. (2008). Divalent metal transporter 1 (DMT1) contributes to neurodegeneration in animal models of Parkinson’s disease. *Proc. Natl. Acad. Sci. U.S.A.* 105 18578–18583. 10.1073/pnas.0804373105 19011085 PMC2587621

[B40] Sanchez M. Galy B. Schwanhaeusser B. Blake J. Bähr-Ivacevic T. Benes V. (2011). Iron regulatory protein-1 and -2: transcriptome-wide definition of bindingmRNAs and shaping of the cellular proteome by iron regulatory proteins. *Blood* 118 e168–e179. 10.1182/blood-2011-04-343541 21940823

[B41] Schneider S. A. Dusek P. Hardy J. Westenberger A. Jankovic J. Bhatia K. P. (2013). Genetics and Pathophysiology of Neurodegeneration with Brain Iron Accumulation (NBIA). *Curr. Neuropharmacol.* 11 59–79. 10.2174/157015913804999469 23814539 PMC3580793

[B42] Tabuchi M. Tanaka N. Nishida-Kitayama J. Ohno H. Kishi F. (2002). Alternative splicing regulates the subcellular localization of divalent metal transporter 1 isoforms. *Mol. Biol. Cell* 13 4371–4387. 10.1091/mbc.E02-03-0165 12475959 PMC138640

[B43] Tabuchi M. Yanatori I. Kawai Y. Kishi F. (2010). Retromer-mediated direct sorting is required for proper endosomal recycling of the mammalian iron transporter DMT1. *J. Cell Sci.* 123(Pt 5) 756–766. 10.1242/jcs.060574 20164305

[B44] Tsuyuki S. Takabayashi M. Kawazu M. Kudo K. Watanabe A. Nagata Y. (2014). Detection of WIPI1 mRNA as an indicator of autophagosome formation. *Autophagy.* 10 497–513. 10.4161/auto.27419 24384561 PMC4077887

[B45] Wang D. Wang L. H. Zhao Y. Lu Y. P. Zhu L. (2010). Hypoxia regulates the ferrous iron uptake and reactive oxygen species level via divalent metal transporter 1 (DMT1) Exon1B by hypoxia-inducible factor-1. *IUBMB Life* 62 629–636. 10.1002/iub.363 20681027

[B46] Wilkinson N. Pantopoulos K. (2014). The IRP/IRE system in vivo: insights from mouse models. *Front. Pharmacol.* 5:176 10.3389/fphar.2014.00176 PMC4112806 25120486

[B47] Zanellati M. C. Monti V. Barzaghi C. Reale C. Nardocci N. Albanese A. (2015). Mitochondrial dysfunction in Parkinson disease: evidence in mutant PARK2 fibroblasts. *Front. Genet.* 6:78 10.3389/fgene.2015.00078 PMC4356157 25815004

[B48] Zorzi G. Nardocci N. (2013). Therapeutic advances in neurodegeneration with brain iron accumulation. *Int. Rev. Neurobiol.* 110 153–164. 10.1016/B978-0-12-410502-7.00008-9 24209438

