# Supplementary figures and images for: Ferrous Iron Up-regulation in Fibroblasts of Patients with Beta Propeller Protein-Associated Neurodegeneration (BPAN)
Source: Front Genet. 2017 Feb 17;8:18. doi: 10.3389/fgene.2017.00018 (PMC5314138; doi:10.3389/fgene.2017.00018)

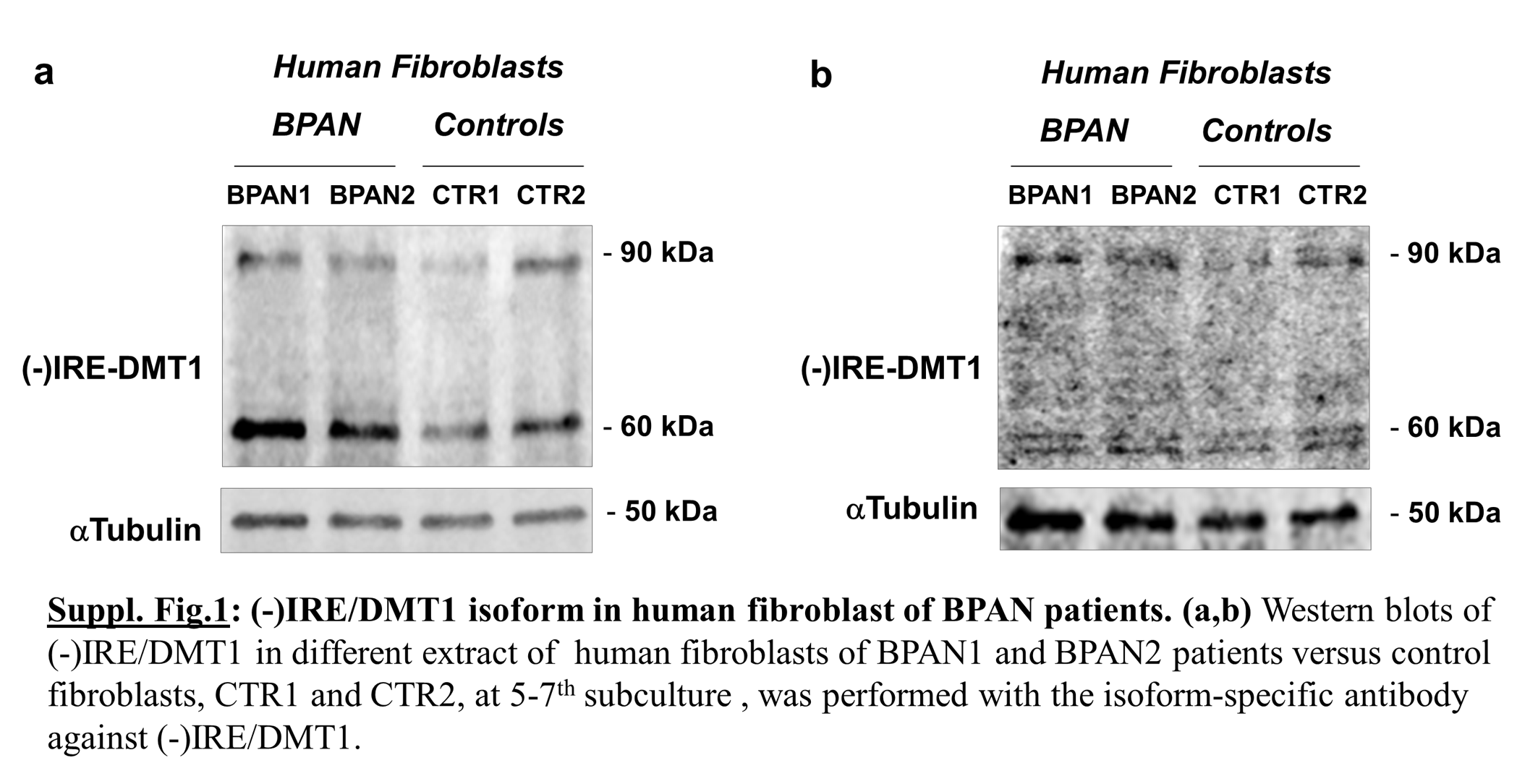

Supplement: Supplementary file 1 [file Image_1.TIF]
